# Role of lymph node dissection in the management of upper tract urothelial carcinomas: a meta-analysis

**DOI:** 10.1186/s12894-018-0336-5

**Published:** 2018-04-10

**Authors:** Runqi Guo, Yuze Zhu, Gengyan Xiong, Xuesong Li, Kai Zhang, Liqun Zhou

**Affiliations:** Department of Urology, Peking University First Hospital and Institute of Urology, National Research Center for Genitourinary Oncology, Beijing, 100034 China

**Keywords:** Lymph node dissection, Recurrence, Survival, Upper urinary tract, Urothelial carcinoma

## Abstract

**Background:**

Lymph node dissection (LND) is not routinely performed during radical nephroureterectomy (RNU) in upper tract urothelial carcinomas (UTUC) and the role of LND has been controversial. We aim to investigate whether patients with LND had improved survival in UTUC patients.

**Methods:**

We performed a systematic literature search of PubMed, Embase, and Cochrane library for citations published prior to January 2016, describing LND performed among UTUC patients and conducted a standard meta-analysis of survival outcomes.

**Results:**

Eleven eligible studies containing 7516 patients satisfied the inclusion criteria. Pooled HRs for cancer-specific survival (CSS) and recurrence-free survival (RFS) were 1.17 (*P* = 0.18) and 1.33 (*P* = 0.19) respectively. However, the patients in the LND group had more advanced tumour stages and grades (*P* < 0.001). Further subgroup analysis showed that among muscle-invasive UTUC patients, the pooled HR for CSS and RFS were 1.10 (*P* = 0.42) and 0.92 (*P* = 0.72) respectively. Besides, no difference was found in CSS and RFS between pN0 and pNx individuals in overall populations and in patients with muscle-invasive UTUC, while pN+ patients had significantly worse prognosis when compared to pN0 patients.

**Conclusions:**

LND during RNU allows more accurate staging and prediction of survival, but it remains uncertain whether LND independently improves survival in patients with UTUC. However, standard use of LND should be further investigated in a multi-center, prospective evaluation to obtain a definitive statement regarding this matter.

## Background

Urothelial carcinomas are the fourth most common tumors [1]. However, upper tract urothelial carcinomas (UTUC) are comparatively uncommon compared to bladder cancer and occupy only 5–10% of urothelial carcinomas [2, 3]. Approximately 30% of patients suffered from muscle-invasive UTUC at presentation and the incidence of lymph node metastasis ranges from 30% to 40% at surgery [4, 5].

Radical nephroureterectomy (RNU) with bladder cuff resection and regional lymph node dissection (LND) is the backbone of management [3, 6, 7]. Generalizing results from previous bladder cancer researches [8–13], it seems reasonable to believe that LND in conjunction with RNU may provide not only utile staging and prognostic information but also a therapeutic benefit in selected patients with UTUC. Nevertheless, the therapeutic benefit of LND in improving survival remains controversial [14–16].

For these reasons, we systematically reviewed the published studies and performed a meta-analysis of studies in which data were reported for the treatment of LND to assess whether patients who achieved LND had improved cancer-specific survival (CSS) or recurrence-free survival (RFS) compared with patients who did not achieve LND, as a means for providing data for standardizing the indication of LND and assisting in creating a better management strategy for UTUC.

## Methods

### Search strategy

We systematically reviewed PubMed, Embase, and Cochrane library for citations published prior to January 2016, describing LND performed among patients with UTUC. The search strategy included the terms: lymphadenectomy or lymph node excision or lymphatic metastases, and upper tract urothelial neoplasms or upper tract urothelial cancer or transitional cell carcinoma of the upper urinary tract. Two authors independently reviewed article titles and abstracts for eligibility, and divergences were settled by consensus.

### Inclusion and exclusion criteria of trials

Studies were included if they met all the following criteria: (1) prospective randomized studies or well-designed non-randomized controlled experiments; (2) studies analyzing the relationship between LND and UTUC prognosis; (3) clearly described outcome assessment by representing it in CSS or RFS; (4) sufficient survival information with hazard ratios (HR) and corresponding 95% confidence interval (CI), or Kaplan–Meier curves comparing survival among pathologic subgroups that were stratified according to LN status (pN0, negative node; pNx, skipping LND; pN+, positive node) or between LND and NLND; and (5) demographics and pathologic characteristics of patients were stratified according to LN status or according to the presence or absence of LND. Studies were excluded if they met one of the following criteria: (1) the article was a review or meta-analysis; (2) No available data could be able to extracted from the previously published studies; (3) the article deal with recurrent UTUC, metastatic carcinoma, previous or concurrent invasive bladder tumors or neoadjuvant chemotherapy; and (4) (potentially) overlapping study populations were reported for the same outcome.

### Data extraction

All studies identified were independently reviewed by two reviewers. Titles and abstracts were screened for initial inclusion and final agreement on study inclusion was made by discussion and consensus with other authors. Two reviewers extracted data from all the included studies independently. Divergences were settled through consensus.

### Quality assessment

The quality of the cohort studies was evaluated using the modified Newcastle-Ottawa Scale, which met the demands of this study [17]. This scale assesses risk in three domains: patient selection, comparability of LND and NLND groups and assessment of outcome (Table 1). To compare the two cohorts, we concentrated on the following variables that had been identified as independent predictors in previous multivariate studies: age, gender, tumor grade and tumor stage [18–21]. Each study was assessed by two reviewers independently. Any divergences were settled by discussion or through arbitrament by a third reviewer if no agreement could be reached.

**Table 1:** Newcastle-Ottawa quality assessment scale

Check list
Selection
•How representative was the control group (lymph node dissection) in comparison with the general elderly population for transitional cell carcinoma of the upper urinary tract? (if yes, one point; no point, if the patients were selected or selection of group was not described)
•How representative was the research group (non-lymph node dissection) in comparison with the elderly population for transitional cell carcinoma of the upper urinary tract? (if data from the same community as the control group, one point; no point, if drawn from a different source or selection of group was not described)
•Assignment for treatment: any detail report? (if yes, one point)
Comparability
•Group comparable for the grade of tumor, clinical TNM staging system (if yes, two points; one point was assigned, if one of these two characteristics had differences; no star was assigned, if the two groups differed)
•Group comparable for age, gender (if yes, two points; one star was assigned, if one of these two characteristics had differences; no point was assigned, if the two groups differed)
Outcome assessment
•Comprehensively evaluated the outcome? (yes, one point for information ascertained by record or International Classification of Diseases; no point, if this information was not reported)
•Adequacy of follow-up (one star, if follow-up > 90%)

### Data analysis and synthesis

We use log HR and the variance as the summary outcome measure from all trials in the meta-analysis. For each trial, HR with the 95% CI of the survival rate was derived and calculated using either the fixed-effects model or the random-effects model [22]. Chi-square test was used to assess the heterogeneity between studies. For *P* values less than 0.1, the assumption of homogeneity was deemed invalid. Therefor, we calculated pooled estimates using random effects modeling, which provides more conservative estimates than fixed effects modeling when heterogeneity was present.

Publication bias is considered as a concern for meta-analyses. In our study, publication bias was assessed by funnel plots and Egger’s regression [23]. Review manager version 5.3 (Cochrane Collaboration, Oxford, UK) was used for data analysis. A *P* value of less than 0.05 was considered statistically significant.

## Results

### Study identification and quality assessment

**Fig. 1:**
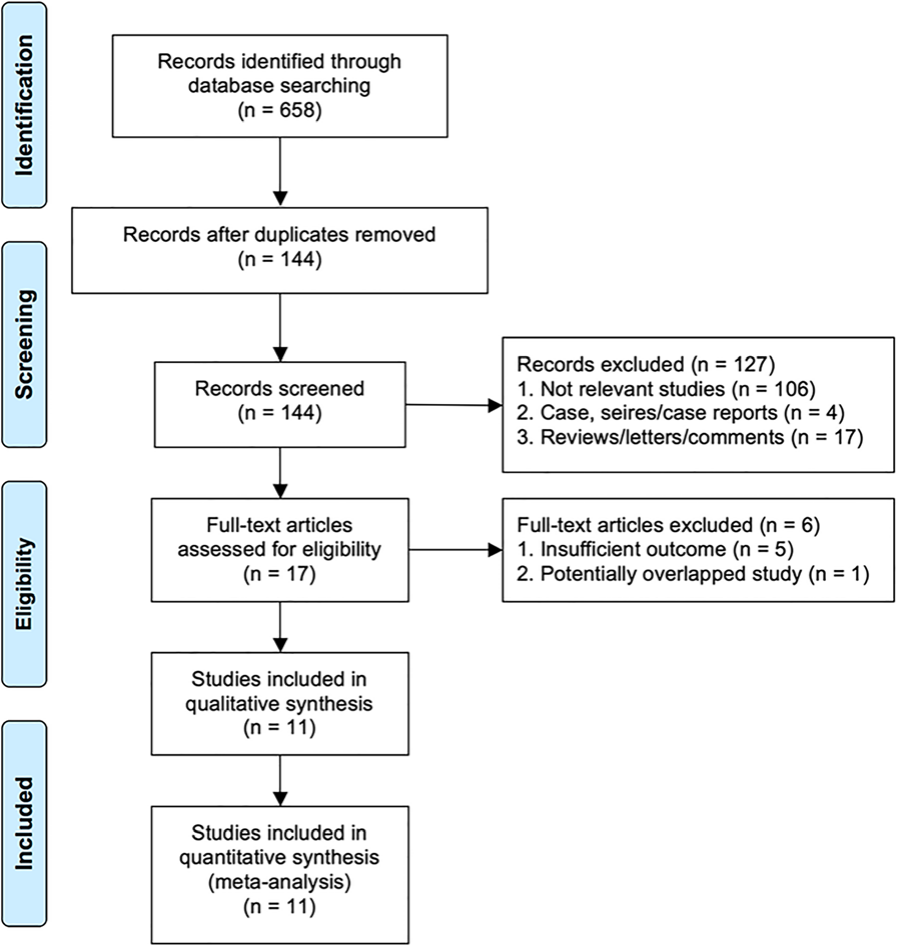
Flowchart of study selection

A total of 658 studies were identified. After excluding duplicates, 144 articles remained, 127 of which were excluded: 106 were apparent irrelevant studies, 4 were case, series/case reports, and 17 were letters/reviews/comments. 17 were reviewed in depth, and a full examination of the text was performed. Five studies were excluded because of insufficient outcome and one was excluded due to potentially overlapping study populations. At last, 11 studies involving 7516 UTUC patients were included into this meta-analysis [14, 15, 16, 24–30, 31] (Fig. 1) (Table 2).

**Table 2:** Characteristics of included studies in meta-analysis

Study	Type of study	Gender	Patients (n)	Follow-up median (month)	Median age (year)	Node status (overall)/ LND or NLND	Extent of LND	Tumor location	Pathologic tumor stage	Tumor grade	Outcome
Kondo T et al 2014 [31]	Prospective	Male 112 Female 54	166	23.7	72.4	pN0 69 pNx 86 pN+ 11	Renal pelvis tumor: LN from the renal hilar to the inferior mesenteric artery. Tumor of upper 2/3 ureter: LN from the renal hilar to the aortic bifurcation. Tumor of lower 1/3 ureter: the ipsilateral pelvic LN	Renal pelvis 90 Ureter 76	≤pT1 62 pT2 27 pT3 72 pT4 5	Low 71 High 95	CSS
Ouzzane A et al 2013 [16]	Retrospective	Male 484 Female 228	714	27.0	70.0	pN0 204 pNx 460 pN+ 50	NA	Renal pelvis 388 Pelvis+ureter 90 Multifocal 236	pTa/Tis 209 pT1 168 pT2 74 pT3 224 pT4 39	G1 71 G2 244 G3 399	CSS, RFS
Mason RJ et al. 2012 [15]	Retrospective	Male 654 Female 375	1029	19.8	68.6	pN0 199 pNx 753 pN+ 77	NA	Renal pelvis 538 Pelvis+ureter 213 Ureter 250	pTa/Tis 108 pT1 463 pT2 160 pT3 244 pT4 54	Low 340 High 689	CSS, RFS
Burger M et al 2011 [30]	Retrospective	Male 542 Female 243	785	34.0	68.0	pN0 136 pNx 595 pN+ 54	Hilar & regional LN adjacent to ipsilateral great vessel	NA	pTa 165 pTis 10 pT1 196 pT2 148 pT3 222 pT4 44	G1 100 G2 226 G3 459	CSS, RFS
Abe T et al 2010 [29]	Retrospective	Male 195 Female 98	293	NA	69.2	pN0 130 pNx 141 pN+ 22	Tumor of renal pelvis tumor & upper 2/3 ureter: Hilar & regional LN adjacent to ipsilateral great vessel Lower ureteral tumor: ipsilateral pelvic LN	Renal pelvis 157 Pelvis+ureter 24 Ureter 112	pTa/Tis 53 pT1 66 pT2 56 pT3 101 pT4 17	Low 185 High 108	RFS
Lughezzani et al 2010 [14]	Retrospective	Male 1666 Female 1158	2824	43.0	72.0	pN0 1835 pNx 747 pN+ 242	NA	Renal pelvis 1913 Ureter 911	pT1 867 pT2 500 pT3 584 pT4 873	G1 156 G2 935 G3 1234 G4 499	CSS
Roscigno M et al 2009 [28]	Retrospective	NA	1130	45.0	69.1	pN0 412 pNx 578 pN+ 140	Renal pelvis and proximally ureteral tumor: LN from the renal hilar to the inferior mesenteric artery Mid and lower ureteral tumors: LN from the renal hilar to the bifurcation of the common iliac artery and ipsilateral pelvic LN	NA	pT1 317 pT2 269 pT3–4544	Low 291 High 839	CSS
Kondo T et al. 2007 [27]	Retrospective	Male 113 Female 56	169	37.3	67.5	LND 81 NLND 88	Renal pelvis tumor: LN from the renal hilar to the inferior mesenteric artery Tumor of upper 2/3 ureter: LN from the renal hilar to the aortic bifurcation Tumor of lower 1/3 ureter: ipsilateral pelvic LN	Renal pelvis 100 Upper ureter 9 Midureter 20 Lower ureter 40	≤pT1 45 pT2 34 pT3 79 pT4 9	NA	CSS
Secin FP et al. 2007 [26]	Retrospective	Male 166 Female 86	252	37.2	69.0	pN0 105 pNx 119 pN+ 28	NA	NA	pTa 71 pTis 12 pT1 46 pT2 35 pT3 72 pT4 8	G1 64 G2 38 G3 143	CSS
Brausi MA et al 2007 [25]	Retrospective	Male 59 Female 23	82	64.7	LND 67.8 NLND 67.1	LND 40 NLND 42	Renal pelvis and upper ureteral tumor: LN from the renal hilar to the inferior mesenteric artery Mid ureteral tumors: LN from the renal hilar to the bifurcation of the common iliac artery Lower ureteral tumor: ipsilateral pelvic LN	Renal pelvis 47 Pelvis+ureter 7 Ureter 28	pT2 38 pT3 36 pT4 8	G2 44 G3 38	CSS
Miyake H et al.*.* 1998 [24]	Retrospective	Male 53 Female 19	72	49	LND 64 NLND 67	LND 35 NLND 38	Renal pelvis and upper ureteral tumor: LN from the renal hilar to the inferior mesenteric artery Mid ureteral tumor: LN from the renal hilar to the bifurcation of the common iliac artery Lower ureteral tumor: ipsilateral pelvic LN	Renal pelvis 40 Pelvis+ureter 3 Ureter 29	pTa 11 pT1 25 pT2 18 pT3 14 pT4 4	G1 12 G2 33 G3 37	CSS

*LND* Lymph node dissection, *NLND* Non-LND, *NA* Not available, *pN+* Positive lymph node, *pN0* Negative lymph node, *pNx* Not undergo lymph node dissection, *CSS* cancer-specific survival, *RFS* recurrence-free survival

The quality assessment of included cohort studies was performed using the modified Newcastle-Ottawa Scale. Studies that scored > 7 were considered as having low risk of bias, scores of 5–7 indicated moderate risk of bias, and scores of < 5 indicated high risk of bias, and the total scores are shown in Table 3. Most studies were deemed to be of moderate risk of bias and we only scored 3 of 11 studies as having low risk of bias. Commonly identified concern was the comparability of LND and NLND groups, especially regarding tumor grade and TNM staging.

**Table 3:** Assessment for quality of included studies

Study	Selection			Comparability		Outcome assessment		Score
	1	2	3	1	2	1	2	
Kondo T et al 2014 [31]	1	1	1	2	0	1	1	7
Ouzzane A et al 2013 [16]	1	1	1	0	2	1	1	7
Mason RJ et al 2012 [15]	1	1	1	0	1	1	1	6
Burger M et al 2011 [30]	1	1	1	0	1	1	1	6
Abe T et al 2010 [29]	1	1	1	0	2	1	1	7
Lughezzani et al 2010 [14]	1	1	1	0	2	1	1	7
Roscigno M et al 2009 [28]	1	1	1	0	1	1	1	6
Kondo T et al 2007 [27]	1	1	1	1	2	1	1	8
Secin FP et al 2007 [26]	1	1	1	1	1	1	1	7
Brausi MA et al 2007 [25]	0	1	1	2	2	1	1	8
Miyake H et al 1998 [24]	1	1	1	2	1	1	1	8

### Meta-analysis results

#### Cancer-specific survival

**Fig. 2:**
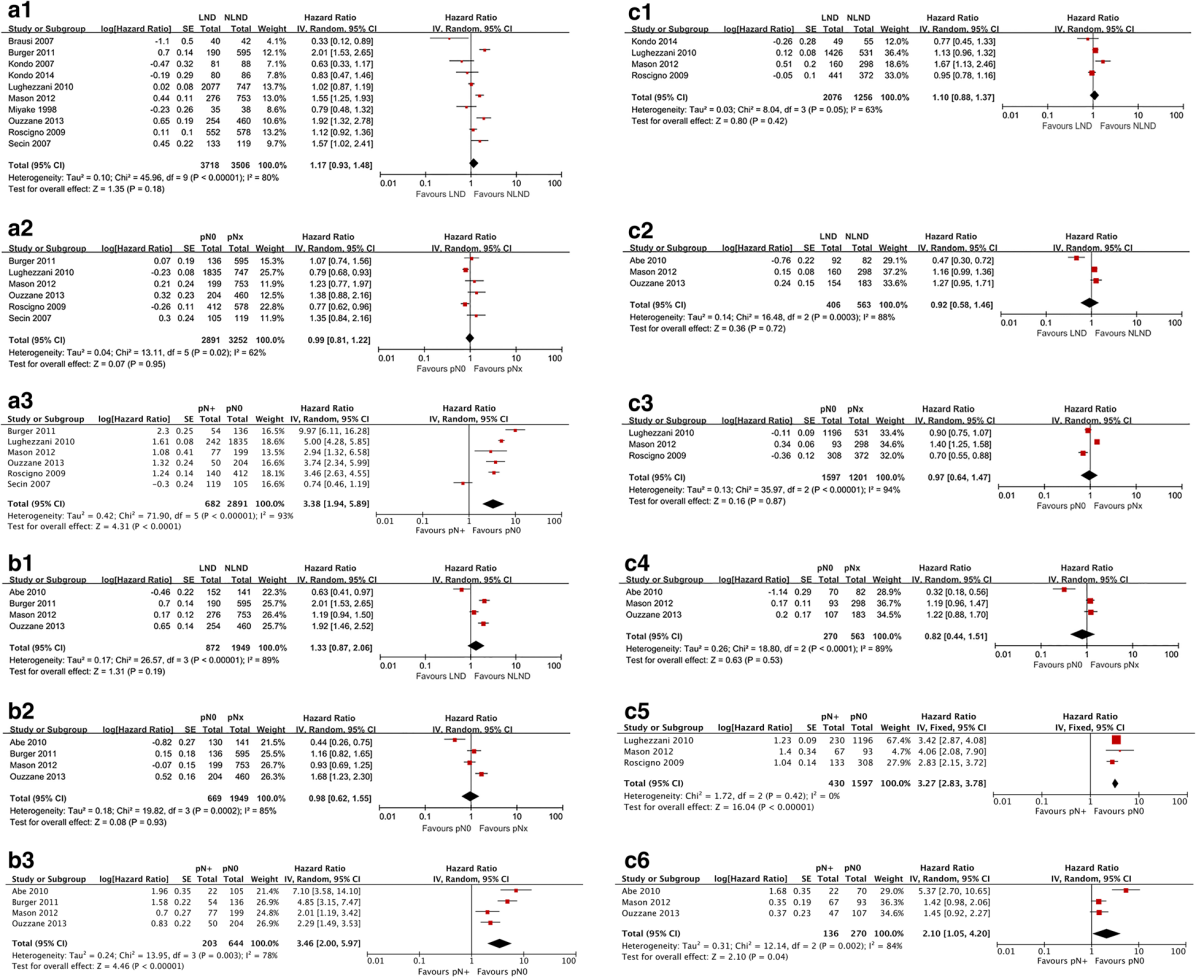
Forest plot comparing survival and subgroup analysis of different pT statuses. (**A1**) CSS in patients receiving LND versus NLND; (**A2**) CSS in patients considered pN0/pNx; (**A3**) CSS in patients considered pN+/pN0; (**B1**) RFS in patients receiving LND versus NLND; (**B2**) RFS in patients considered pN0/pNx; (**B3**) RFS in patients considered pN+/pN0; (**C1**) CSS in muscle-invasive UTUC patients receiving LND versus NLND; (**C2**) RFS in muscle-invasive UTUC patients receiving LND versus NLND; (**C3**) CSS in patients of muscle-invasive UTUC considered pN0/pNx; (**C4**) RFS survival in patients of muscle-invasive UTUC considered pN0/pNx; (**C5**) CSS in patients of muscle-invasive UTUC considered pN+/pN0; (**C6**) RFS survival in patients of muscle-invasive UTUC considered pN+/pN0. CSS, cancer-specific surviva; LND: lymph node dissection; NLND: non-lymph node dissection; pN0: Negative lymph node; pNx: Not undergo lymph node dissection; RFS, recurrence-free survival; UTUC: upper tract urothelial carcinoma.

Of the 10 studies that referred to CSS, there was significant heterogeneity among them (*I*^*2*^ = 80%, *Chi*^*2*^ = 45.96, *P* < 0.00001). Thus, a random-effects model was used to calculate the pooled HR and corresponding 95% CI. No statistically significance was found between the LND group and the NLND group (HR = 1.17, 95% CI: 0.93–1.48, *P* = 0.18) (Fig. 2 A1). Besides, patients with pN0 did not have better CSS compared with those with pNx (HR = 0.99, 95% CI: 0.81–1.22, *P* = 0.95) with significant heterogeneity (*I*^*2*^ = 94%, *Chi*^*2*^ = 35.97, *P* < 0.00001) (Fig. 2 A2), while patients with pN+ showed poor CSS compared with those with pN0 (HR = 3.38, 95% CI: 1.94–5.89, *P* < 0.0001) with significant heterogeneity (*I*^*2*^ = 93%, *Chi*^*2*^ = 71.90, *P* < 0.00001) (Fig. 2 A3).

To explore the source of apparent heterogeneity, we compared the differences in tumor stage and tumor grade between the groups, thereby demonstrating the features between the groups using Chi-square tests and Fisher’s exact tests for categorical variables (Table 4). The results showed that there was remarkable significant difference in tumor stage and tumor grade between the LND group and NLND group (*P* < 0.001), which might have significant association with the heterogeneity.

**Table 4:** Chi-square tests for two groups

Variable	LND (n, %)	NLND (n, %)	*P* value
Tumor stage			< 0.001
≤T1	1210 (31.3)	1684 (46.2)	
T2	722 (18.7)	637 (17.5)	
T3	1204 (31.2)	990 (27.2)	
T4	726 (18.8)	335 (9.2)	
Tumor grade			< 0.001
Low grade or ≤ G2	1610 (35.4)	1281 (43.2)	
High grade or > G2	2936 (64.6)	1682 (56.8)	

*LND* lymph node dissection, *NLND* non LND

#### Recurrence-free survival

Significant heterogeneity was observed in the four studies that focused on RFS (*I*^*2*^ = 89%, *Chi*^*2*^ = 26.57, *P* < 0.00001), hence we utilzed the random-effects model. The pooled HR for RFS was 1.33 (95% CI: 0.87–2.06, *P* = 0.19), which indicate that LND was not associated was better RFS in patients with UTUC (Fig. 2 B1). Meanwhile, in consideration of pN0/pNx, no significant difference in RFS between pN0 and pNx was found (HR = 0.98, 95% CI: 0.62–1.55, *P* = 0.93) and there was significant heterogeneity among them (*I*^*2*^ = 85%, *Chi*^*2*^ = 19.82, *P* = 0.0002) (Fig. 2 B2). In contrast, pN+ showed poor RFS compared with those pN0 (HR = 3.46, 95% CI: 2.00–5.97, *P* < 0.0001) with significant heterogeneity (*I*^*2*^ = 78%, *Chi*^*2*^ = 13.95, *P* < 0.003) (Fig. 2 B3).

#### Subgroup analysis

We performed subgroup analysis according to pT statuses, among patients with muscle-invasive UTUC. Data for CSS in patients with muscle-invasive UTUC were reported in four studies, and there was heterogeneity among those studies (*I*^*2*^ = 63%, *Chi*^*2*^ = 8.04, *P* = 0.05); hence, we utilzed the random-effects model. However, no statistically significance was found between the two groups (HR = 1.10, 95% CI: 0.88–1.37, *P* = 0.42) (Fig. 2 C1).

Additionally, the results of the subsequent analyses showed no difference in RFS between the LND group and the NLND group among muscle-invasive UTUC individuals (HR = 0.92, 95% CI: 0.58–1.46, *P* = 0.72) and there was relatively high heterogeneity in this subgroup (*I*^*2*^ = 88%, *Chi*^*2*^ = 16.48, *P* = 0.0003) (Fig. 2 C2).

Furthermore, among the patients with muscle-invasive UTUC, no significant difference between pN0 and pNx was found in CSS and RFS (HR = 0.97, 95% CI: 0.64–1.47, *P* = 0.87; and HR = 0.97, 95% CI: 0.64–1.47, *P* = 0.87, respectively) and there was significant heterogeneity (*I*^*2*^ = 94%, *Chi*^*2*^ = 35.97, *P* < 0.00001; and *I*^*2*^ = 89%, *Chi*^*2*^ = 18.80, *P* < 0.00001, respectively) (Fig. 2 C3 & 2C4). However, patients with pN+ showed poor CSS and RFS in comparison with those with pN0 (HR = 3.27, 95% CI: 2.83–3.78, *P* < 0.00001; and HR = 2.10, 95% CI: 1.05–4.20, *P* = 0.0002, respectively) (Fig. 2 C5and C6).

### Publication bias

**Fig. 3:**
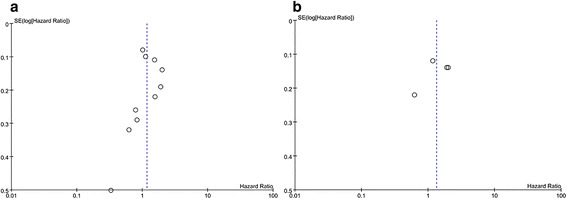
Funnel plot for the evaluation of potential publication bias. (**a**) cancer-specific survival; (**b**) recurrence-free survival

The publication bias was detected using a funnel plot of the meta-analysis result. The basic symmetry of the funnel plots suggested that there was no obvious publication bias (Fig. 3). The Egger’s test for CSS and RFS did not show any evidence of publication bias.

## Discussion

Radical cystectomy with pelvic LND for muscle-invasive bladder cancer is relatively standardized because it improves tumor staging and survival of patients [32, 33]. However, potential benefit of LND during RNU on survival for UTUC is still controversial [15, 30]. On the basis of the latest European guidelines on UTUC, LND should be performed in conjunction with RNU not only for better tumor staging but also for prognosis improvement [3]. Nevertheless, this recommendation is only Level III evidence. Thus, we reviewed the published studies and conducted a meta-analysis to clarify the prognostic value of LND in patients with UTUC.

In the present research, 11 studies were eligible and the HRs of cumulative survival rates were summarized quantitatively. Our analysis revealed that pN+ patients had significantly worse prognosis when compared to pN0 patients. The same results were observed when restricting the analyses to patients with muscle-invasive carcinomas, who should, anyway, be systematically considered for staging LND in light of this growing body of data.

However, no difference was found in survival or disease recurrence when comparing pN0/pNx individuals and the LND/NLND groups. The sample size of the included studies could explain these results. Most of the early years studies include small numbers of patients (less than 200), while larger series (more than 1000) with more events only emerged recently. Besides, the decision to perform LND was left to the discretion of the surgeon, it is possible that those NLND patients had less aggressive disease than LND patients, and that a true benefit to LND does exist. An increased risk of cancer-related death is usually related to higher tumor stage and grade. In this comparison, there was no significant difference in CSS and RFS between the LND group and the NLND group, which may reversely suggest the possible therapeutic value of LND for patients with more aggressive tumors. Nevertheless, the results remained no significant difference when controlling for tumor stage. Conversely, in a review by Kondo and Tanabe [34], it was highlighted that when the regional nodes were completely dissected, the patients with the advanced stage had significantly higher survival compared with those without LND.

Interestingly, pNx was not associated with poor CSS and RFS in patients with muscle-invasive carcinomas and in overall population. Several explanations may account for our results. First, pNx individuals were most likely identified by their surgeons as low risk for nodal metastases. It is also possible that pNx individuals may harbor micrometastatic lymph node deposits, which could be either destroyed or removed during the surgery, without being identified as pN1 by the pathologist. Furthermore, the lack of standardized anatomical limits and indication for the LND could account for our results: some patients certainly had very limited dissection and unsuitable for tumor location, leading to a wrong histological report of pN0 stage even though they had nodal metastasis not including in the LND.

It is noteworthy that 49.0% RNU patients were staged as pNx in our studies. In 2009, Roscigno et al. pointed out that patients with pN0 disease had a better prognosis than pNx disease in patients with muscle-invasive carcinomas [28]. It is conceivable that, despite a higher pNx rate at tertiary care centers, the extent of LND in those in whom it was performed was substantially greater than the LND extent in the community. Under this premise, a more important stage migration towards true pN0 status may have occurred at tertiary care centers than in the current population-based series [14]. Taken together, our findings suggest that pNx individuals have no better prognosis than pN0 individuals.

Without standardized criteria for who should receive LND and how extensive LND should be, comparisons between series proved to be challenging. It was reported that the patients with incomplete LND in showed lower survival than those with complete LND, which reached statistical significance. Five-year CSS in the patients with pT2 or higher and pT3 or higher was 77.9% and 73.2% in the patients with complete LND, but just 54.0% and 43.7% in those with incomplete LND and 59.0% and 47.3% in those with NLND [34].

The most important finding of our study is that LND patients had no worse prognosis than NLND patients, especially in those with muscle-invasive carcinomas. According to a recent review, carrying out LND for UTUC is unlikely to be time-consuming and to increase the risk of major complications [34]. Although the current quality of evidence is generally not high, which may lead to biased and uncertain results, it might still suggest that the role of LND in UTUC is of importance, as UTUC is likely to simulate the biological behavior of bladder cancer because of the same histology among the two diseases.

Limitation should also be considered. First, the sources of the publications were limited, thus potentially introducing inevitable publication bias. Second, although 11 eligible studies involving 7516 patients were included in this meta-analysis, most of them were retrospective studies and the sample sizes of some selected studies are small, which might render the results less reliable. Third, marked heterogeneity of studies was seen in pooled-analysis of CSS and RFS. Furthermore, 7 of the 11 included studies provided the extent of LND, but the indication and extent of LND were not standardized. Last but not the least, as the included studies spanned a 10-year interval, the year in which the surgery occurred could be associated with different survival rates due to better imaging, earlier diagnosis and improved peri-operative strategies of care. In the future, the role of LND should be further examined by validating templates of regional lymph nodes, and by prospective studies with larger numbers of patients. Then, we will discuss whether LND can be a standard treatment for UTUC.

## Conclusions

LND during RNU allows more accurate staging and prediction of survival, but it remains uncertain whether LND independently improves survival in patients with UTUC. However, standard use of LND should be further investigated in a multi-center, prospective evaluation to obtain a definitive statement regarding this matter.

## Abbreviations

CI: Confidence interval
CSS: Cancer-specific survival
HR: Hazard ratios
LND: Lymph node dissection
RFS: Recurrence-free survival
RNU: Radical nephroureterectomy
UTUC: Upper tract urothelial carcinomas
